# Economic burden of preemptive treatment of CMV infection after allogeneic stem cell transplantation: a retrospective study of 208 consecutive patients

**DOI:** 10.1186/s12879-017-2854-2

**Published:** 2017-12-05

**Authors:** Christine Robin, François Hémery, Christel Dindorf, Julien Thillard, Ludovic Cabanne, Rabah Redjoul, Florence Beckerich, Christophe Rodriguez, Cécile Pautas, Andrea Toma, Sébastien Maury, Isabelle Durand-Zaleski, Catherine Cordonnier

**Affiliations:** 1 Department of Hematology, Assistance Publique-Hopitaux de Paris (APHP), Henri Mondor Teaching Hospital, 94010 Créteil, France; 2 University Paris-Est Créteil (UPEC), 94000 Créteil, France; 3 Department of Medical Information, APHP, Henri Mondor Teaching Hospital, Créteil, France; 4 URC-Eco APHP ECEVE UMRS 1123, Paris, France; 5 Department of Virology, and INSERM U955 Team 18, APHP, Henri Mondor Teaching Hospital, 94000 Créteil, France; 6 Department of Public Health, APHP, Henri Mondor Teaching Hospital, 94000- Créteil, France

**Keywords:** Allogeneic stem cell transplantation, CMV infection, Antivirals, Costs

## Abstract

**Background:**

Cytomegalovirus (CMV) infection and disease (CMV episodes) are global concerns after allogeneic hematopoietic stem cell transplantation (HSCT). They affect survival, both by direct and indirect effects. Due to safety issues of current anti-CMV antivirals, long-term CMV prophylaxis is poorly tolerated and the most common strategy to decrease the incidence of CMV disease is preemptive. New, less toxic, molecules are currently being assessed for CMV prophylaxis which should replace or considerably decrease the preemptive approach. The aim of this study was to assess the economic burden of CMV episodes after HSCT with a preemptive approach.

**Methods:**

We analyzed data from 208 consecutive adults transplanted in our institution, between 2008 and 2013. Hospital resource utilization was retrieved via the linked hospital admissions and Diagnostic Related Groups for the period of conditioning to 12 months after transplant.

**Results:**

CMV episodes occurred in 70 patients (34%) over the first 12 months following HSCT, after a mean of 75 days (median: 46 (7–334)). The mean total length of stay was significantly associated with the occurrence of a CMV episode (113.9 vs. 87.5 days, *p* = 0.0002) but was associated neither with the pre-transplant CMV serology of donors/recipients nor with survival. The mean cost of transplant was €104,016 (SD = €37,281) after 12 months. Bivariate and multivariate analyses indicated that the occurrence of >1 CMV episode increased the costs of allogeneic HSCT by 25–30% (*p* < 0.0001).

**Conclusion:**

Our study, which is the largest, single-institution cost study of allogeneic HSCT in Europe, shows that two or more CMV episodes significantly increased the transplant cost. New prophylactic strategies to prevent CMV infection and disease should decrease transplant costs.

## Background

Cytomegalovirus (CMV) infection and diseases are global concerns for the transplant community after allogeneic hematopoietic stem cell transplantation (HSCT). CMV-seropositive recipients of HSCT have a higher rate of mortality than CMV-seronegative patients, especially in transplants from unrelated or mismatched donors, and even more if the donor is CMV-seronegative [1, 2]. In agreement with this finding, the occurrence of CMV reactivation before day 100 is associated with higher non-relapse mortality [3]. There are many mechanisms by which the CMV may affect survival, including CMV infection and disease, but also indirect effects relating to graft-versus-host disease (GVHD), side-effects of antiviral drugs, and concomitant non-viral infections [2]. Preemptive treatment of CMV infection is currently the most common strategy to reduce the risk of CMV disease and the survey of Pollack et al. showed that the CMV practices are not different between North America and Europe [4]. This preference of many programs for preemptive over prophylactic treatment is mainly because of the side effects of the most common antiviral drugs (ganciclovir and foscavir) available to treat CMV. Indeed, these drugs are not safe enough to be routinely given for several weeks [1]. In three randomized trials to evaluate early prophylactic ganciclovir use after allogeneic HSCT, the benefit of preventing CMV infection, and possibly CMV disease, was offset by a greater risk of bacterial or fungal infections due to neutropenia [5–7]. Long-term administration of foscavir also raises safety issues, exposing patients to unacceptable renal toxicity [8].

New anti-CMV drugs such as maribavir [9], brincidofovir [10], letermovir [11], and CMV vaccines [12, 13] are currently being, or have been recently evaluated in phase 2 or 3 studies. On many aspects, the new antiviral drugs have safer profiles than available anti-CMV drugs, and the results of the ongoing trials may encourage CMV prophylaxis after HSCT [1], especially with letermovir which has been shown, in a large placebo-controlled phase 3 study, to decrease CMV infection in CMV seropositive patients [14]. However, considering a minimal 3-month duration of the at-risk period for CMV reactivation, these prophylactic approaches will be probably more expensive than the actual preemptive approach whose duration of treatment is usually limited to 2–4 weeks.

In this study, we aimed to evaluate the overcost related to CMV infection and disease over the first year of transplant, using a preemptive strategy, and to know whether the transplant cost can be predicted from the CMV serological status of the donor and recipient.

## Methods

### Study design

This was a retrospective study of all consecutive patients who received a first allogeneic HSCT in our department between January 2008 and December 2013. We hypothesized that CMV serological status of patients and donors and subsequent CMV infections and diseases are cost predictors/drivers after transplant. The primary objective of our study was to assess the over cost possibly related to CMV infection and disease, and test whether donor and recipient CMV serologies, are predictors of 12-month costs. The secondary objective was to evaluate if donor and/or recipient CMV serology, infection, or disease independently affects the duration of hospitalization, and stay in the intensive care unit (ICU). Medical data were collected from the original charts. Hospital resource utilization was retrieved via the linked hospital admissions and Diagnostic Related Groups (DRG) for the period of conditioning to 12 months after transplant. Patients who received a second transplant were censored from the study on the date of the 2nd transplant. Before the transplant, all patients gave their written consent for their data be collected after the transplant. According to the French Health Public Law (CSP Art L1121–1.1), such an investigation does not require specific informed consent or ethics committee approval.

### Clinical definitions and procedures

GVHD was diagnosed and graded according to established criteria [15–17]. The myeloablative conditioning regimens were defined according to the EBMT guidelines [18]. Non-myeloablative conditioning regimens were divided into two categories: fludarabine with 2 Gray total body irradiation (‘mini-conditioning’) and all other regimens (‘reduced intensity’). CMV infection and disease were assessed according to Ljungman and Paya, 2002 [19]. CMV infection was defined as either a pp65 antigenemia ≥5 positive/200000 cells or a blood qPCR ≥1000 copies/mL (equivalent to 745 IU/mL) once.

### Cost analysis

Costs were estimated from the perspective of the third party payer, which in France covers all medical costs for patients with severe chronic condition. Inpatient and outpatient charges were obtained from the hospitals’ billing systems using the DRG codes starting from the time of admission for the transplant to 12 months post-transplantation. The costs of donor identification and graft procurement were excluded as they would not be affected by CMV infection. Out-of-hospital costs were excluded under the assumption that any event of importance would result in an admission. Consequently, costs of out-patient antiviral treatment were not recorded. We used patient-level record linkage to ensure that all data was retrieved and used. The length of stay was computed for the index and subsequent admissions; we identified transfers to the intensive care unit. Outpatient admissions were attributed to a length of stay of one day. All costs were adjusted to 2015 tariffs. Data collection was anonymous.

### Statistical analysis

Tests for the hypothesis of no difference across groups were performed using Student tests and ANOVA when appropriate, chi2 tests, and the Wilcoxon signed rank test. To identify the pre- and post-transplant factors that influence costs during the first year, we performed a bivariate analysis to identify the relevant variables. This was followed by multiple linear regression modeling, which included baseline patient and transplant characteristics that had been found to significantly influence cost. We excluded variables that were highly inter-correlated and therefore could not be entered in one single model. We selected two models which included: patient age, sex, conditioning, number of CMV episodes, graft match, and survival. Because age, conditioning, graft match, and survival were highly correlated, they were tested separately in the models. We used a non-normal (gamma) distribution that best fitted costs to adjust for right-skewed data. Results are reported as the mean and standard deviation (SD) or median and interquartile range (IQ) as appropriate. All costs are in 2015 € (1US $ = 0.83 € by the OECD purchasing power parity index). Analyses were performed using Excel (2010, Microsoft) and SAS (9.3, SAS corp. NC) software.

## Patients

The study included 208 consecutive adult patients; of these, six were censored at the time of second transplant. The median follow-up was 18 months. The characteristics of the patients are summarized in Table 1. Most patients received transplants because of acute leukemia. The graft was from an HLA-identical sibling (40%), an unrelated donor (51%) or a cord-blood unit (9%). The CMV donor/recipient serologies are shown in Table 2. One hundred and thirty recipients (62%) were CMV seropositive.

**Table 1:** Patient characteristics and bivariate analysis of the costs of the first 12 months after allogeneic stem cell transplantation

Variable	Characteristics	Cost after 12 months (€)		
		N	Mean (SD)	*P*-value
Age (years)	< 30	31	103,146 (44,270)	0.6285^a^
	30 to 50	65	107,689 (35,434)	
	> 50	112	102,125 (36,422)	
Sex	F	87	99,375 (33,651)	0.1282^b^
	M	121	107,353 (39,485)	
Underlying disease	Acute leukemia and myelodysplastic syndrome	143	100,960 (31,157)	0.2259^a^
	Lymphoproliferative disorder	46	111,367 (48,575)	
	Myeloproliferative disorder	12	116,491 (56,018)	
	Other	7	96,756 (22,709)	
Donor type	HLA identical sibling	83	103,795 (41,940)	0.8294^a^
	Unrelated donor	107	104,998 (33,467)	
	Cord blood unit	18	99,200 (37,742)	
D/R match	HLA identical sibling	83	103,795 (41,940)	0.8986^a^
	Unrelated donor 10/10 and cord blood unit 6/6	63	102,644 (32,594)	
	Unrelated donor 9/10 and cord blood unit 4/6 or 5/6	62	105,706 (35,598)	
Stem cell source	Bone marrow	50	99,679 (39,183)	0.7950^a^
	Peripheral blood stem cell	140	106,184 (36,604)	
	Cord blood unit	18	99,200 (37,742)	
Conditioning regimen	Reduced intensity	92	108,178 (31,779)	0.3566^a^
	Mini	61	101,129 (42,830)	
	Myeloablative	55	100,256 (39,172)	
Recipient CMV serology	R+	130	103,494 (34,279)	0.7950^b^
	R-	78	104,886 (42,030)	
Number of CMV episodes	0	138	99,793 (39,654)	0.0214^a^
	1	44	104,815 (24,271)	
	≥2	26	125,080 (36,290)	
Acute graft-versus-host disease	0 and 1	112	96,278 (37,441)	0.0011^a^
	2 to 4	96	113,043 (35,180)	
Chronic graft-versus-host disease	No	140	101,858 (33,633)	0.2320^b^
	Yes	68	108,458 (43,789)	
Alive at the end of the mentioned period	No	80	109,067 (29,356)	0.1227^b^
	Yes	128	100,859 (41,270)	
First CMV infection	≤100 days	53	109,015 (30,231)	0.1841 ^b^
	>100 days	18	119,834 (32,142)	

^a^Anova test; ^b^ Student test

**Table 2:** Number of patients who developed CMV episode(s) within 12 months of transplant according to donor (D) and recipient (R) CMV serology

Pre-transplant CMV serology	Total number (%)	Number (%) of patients who developed CMV episode(s)		
		0	1	≥2
D−/R-	58 (28%)	55 (26.4%)	1 (0.5%)	2 (1.0%)
D−/R+	59 (28.5%)	28 (13.5%)	19 (9.1%)	12 (5.8%)
D+/R-	20 (9.5%)	15 (7.2%)	4 (1.9%)	1 (0.5%)
D+/R+	71 (34%)	40 (19.2%)	20 (9.6%)	11 (5.3%)
Total	208 (100%)	138 (66.4%)	44 (21.2%)	26 (12.5%)

The patients were managed for CMV risk according to local written procedures, established according to the EBMT [20], and international [21] guidelines. Briefly, patient’s blood, except for CMV seronegative patients transplanted with a seronegative donor, was screened weekly for pp65 Ag (CMV Brite Turbo kit, IQ Products) until September 2009, or plasma CMV DNA (Artus® CMV RG PCR Kit, Qiagen) from October 2009, until day 100 or for longer in cases of persistent immunosuppression. No patient received CMV prophylaxis or prophylactic intravenous immunoglobulins. After discharge following the transplant, all patients were seen in our day-care center at least once a week until day 100, then every two weeks from day 100 to day 180, then monthly, or more often if required. All infected patients received ganciclovir (5 mg/kg × 2/d) or foscavir (60 mg/kg × 2/d) for 14 days, either in the ward or at home, according to their clinical status, blood counts, renal function and the concomitant therapies. Patients who had a viral load below these cut-offs did not receive anti-CMV antiviral drugs. Valganciclovir was not routinely used as first^t^ line treatment of CMV infection. Maintenance treatment of 14 additional days was given at the discretion of the physician but was recommended for cases of severe GVHD and/or transplant from an unrelated donor. Patients with CMV disease were treated with the same doses of ganciclovir as those used to treat CMV infection, but with higher doses of foscavir (90 mg/kg ×2 /d). Patients with CMV pneumonia additionally received intravenous immunoglobulins.

## Results

### CMV infection and disease

Nine patients developed CMV disease (gut disease: 7; pneumonia: 2). Due to this small number of cases, these episodes of CMV disease were pooled with CMV infections, referred to hereafter as “CMV episodes”. Seventy patients (34%) developed at least one CMV episode at 12 months – 88% of these patients developing at least one CMV episode within 6 months after transplant - documented by either pp65 antigenemia (14 episodes) or by qPCR (56 episodes), after a mean time of 75 days (median: 46 (7–334)). Among these 70 patients, 44 patients had experienced one episode and 26 had more than one episode. Three patients of the seronegative donor/seronegative recipient group (*n* = 58) had at least one CMV episode (Table 2). CMV episodes were significantly related to acute GVHD (*p* = 0.0001) and death (*p* = 0.0389).

### Cost of transplant and length of stay

The total average cost for a transplant was €104,016 (SD = €37,281) after 12 months. Ninety percent of hospital resources were used during the first six months of transplant.

The average total length of stay was 96.5 days (SD = 49 days). Forty seven (23%) patients had been admitted to the ICU for an average duration of 10.5 days (SD: 10.8 days, median 6 days, IQ range 2–16 days) over the first year of transplant. Neither CMV donor or recipient serology nor survival were associated with total length of stay or length of stay in the ICU. However, the occurrence of at least one CMV episode was significantly associated with higher total length of stay: 113.9 days (SD = 51.1) for infected patients vs. 87.5 days (SD = 45.5) for non-infected patients (*p* = 0.0002), respectively.

### Relationships between costs, donor/recipient CMV serology and CMV episodes

The results of bivariate analyses for costs are shown in Table 1. Costs were significantly associated with the occurrence of more than one CMV episode (a 25–30% increase depending on the model), after 12 months whereas costs were not associated with the occurrence of one CMV episode or the pre-transplant donor or recipient CMV serology, regardless of the CMV group (D−/R- vs others or R+ vs R- or D−/R- vs D+/R- vs D−/R+ vs D+/R+). The costs were not significantly different in patients who developed their first CMV episode before day 100 and in those who did it after day 100. Acute GVHD ≥ grade 2 significantly increased costs whereas chronic GVHD did not, regardless of the severity.

The results of the multivariate analyses are shown in Table 3. Due to the strong association between acute GVHD and CMV episodes (*p* < 0.0001), acute GVHD was not included in the models. Both donor/recipient match and conditioning were excluded from the model, the former being highly correlated with survival (*p* = 0.0003) and the latter with age (p < 0.0001). No pre-transplant information (age, sex, underlying disease, status, type of donor and of graft, donor and recipient CMV serology) independently predicted cost, except for reduced intensity conditioning regimens which increased costs by 13% relative to myeloablative conditioning. Of the post-transplant clinical events, CMV episodes and death were positively associated with costs at six months (data not shown). At 12 months, only CMV episodes were significantly associated with costs (Table 3).

**Table 3:** Multivariate analyses of costs within the first 12 months following allogeneic HSCT for 208 consecutive patients

Variable	Classes	Estimated value	Wald 95% Confidence Limits		*P* value
Model 1 included age, sex, number of CMV episodes and survival					
Intercept	0	€119,539	€101,265	€141,111	<0.0001
Age (years)	< 30	1.08	0.95	1.22	0.263
	30 to 50	1.07	0.97	1.17	0.1853
	≥ 50	1.00	1	1	0
Sex	F	0.92	0.84	1.01	0.0647
	M	1.00	1	1	0
Number of CMV episodes	0	0.80	0.70	0.91	0.0008
	1	0.84	0.72	0.98	0.0253
	≥2	1.00	1	1	0
Survival status	Dead	1.08	0.99	1.18	0.0939
	Alive	1.00	1	1	0
Model 2 included age, sex, number of CMV episodes, year of transplant and type of graft. Donor/recipient match was excluded because of strong correlation with the type of graft					
Intercept	0	€105,461	€86,051	€129,250	<.0001
Age (years)	< 30	1.07	0.97	1.18	0.1486
	30 to 50	1.06	0.93	1.20	0.3757
	≥ 50	1.00	1.00	1.00	0
Sex	Females	0.93	0.85	1.02	0.13
	Males	1.00	1.00	1.00	.
Number of CMV episodes	0	0.78	0.68	0.90	0.0003
	1	0.83	0.71	0.97	0.0201
	≥2	1.00	1.00	1.00	0
Year of transplant	2008	0.83	0.71	0.97	0.0187
	2009	1.01	0.87	1.18	0.8646
	2010	1.05	0.91	1.22	0.509
	2011	1.07	0.92	1.24	0.3775
	2012	0.97	0.83	1.13	0.6772
	2013	1.00	1.00	1.00	0
Type of graft	HLA-identical sibling	1.14	0.96	1.34	0.1242
	Unrelated donor	1.13	0.96	1.32	0.1384
	Cord Blood Unit	1.00	1.00	1.00	0

We used two models to test explanatory variables that were strongly correlated (e.g age and conditioning)

## Discussion

We show that having two or more CMV episodes after allogeneic HSCT increased the transplant cost of 25–30% whereas having only one episode did not. Additionally, our estimate of the average cumulative 12-month costs of allogeneic HSCT for more than 200 consecutive patients is the largest, single-institution cost study in Europe. The average hospital cost per transplant patient, as paid by the third party payer, was €104,000 and corresponded to a total of 96 days in hospital, and higher costs were significantly driven by the number of CMV episodes.

Despite the use of various costing methods, previous, smaller, cost studies of stem cell transplantation have generated consistent results showing an average 12 month cost of about €100,000, in the same range in the United States [22–25] and in Europe [26]. Eighty percent of this cost is incurred during the first 6 months [22–26]. Few cost predictors have been identified, i.e. pretransplant patient characteristics, preferably those that can be altered by medical intervention. Saito et al. [24] explored cost predictors and found only GHVD prevention among the actionable factors. Lee et al. [23] found no relationship between pre-transplant information and total costs except for mismatched donors. Khera et al. [22] reported graft source, donor type and HLA match as pre-transplant predictors, none of which are easily actionable. In our study, we did not find any pre-transplant predictors of cost, even though we specifically tested CMV D/R serology.

Most multivariate cost models, however, found that GVHD and infections were important cost drivers [22–24, 26, 27]. Consistently, in a previous French study [27], we showed that the main cost drivers were acute grade II-IV GVHD and infections which are usual causes of prolongation of hospitalization or re-hospitalization. However, we did not look specifically at CMV episodes. In fact, few studies have specifically focused on CMV as a cost driver. Pre-transplant CMV serology of the donor or of the recipient has never been found to predict costs significantly, although a trend for lower costs in the seronegative donor/recipient groups has sometimes been found [22, 24]. CMV infection was identified as a cost driver in the 90’s, at a time when no anti-CMV drugs were available and the CMV-related mortality was very high [28]. In a recent US study, patients who received preemptive treatment for CMV infection incurred an additional cost of US $58,000–74,000 [29]. In our study, the occurrence of CMV episodes was significantly associated with higher length of stay and higher costs. Due to the strong association and bidirectional effects between GVHD and CMV [2, 30], both factors increasing the risk of other infections, it is possible that the over cost associated with CMV episodes only reflects the selection of the most severe transplant population. Indeed, it will be a challenge for future anti-CMV prophylactic approaches to be cost-effective by reducing both the direct and indirect effects of CMV, possibly including GVHD.

Until now, most stem cell transplant centers have been using a preemptive strategy for CMV infection [4, 31] with a significant risk of recurrence when CMV-specific immunity is not recovered when antivirals are stopped. However, new anti-CMV compounds have recently or are currently being studied for prophylaxis [9–11]. It may be expected that some of these compounds could prevent the occurrence of CMV episodes, and eventually the indirect effects of CMV. As most of these drugs have safe profiles and are consistent with outpatient management, the transplant community could prefer a prophylactic approach for the first 3 months of transplant to reduce hospitalization. By showing that the occurrence of two or more CMV episodes increases the cost of a transplant by 25–30% (€25–30,000), our study gives a reasonable assessment of the costs which could be saved if any efficient CMV prophylaxis would dramatically decrease CMV episodes. Roughly half (*n* = 70) of our 130 seropositive patients developed a CMV episode. If new CMV prophylactic treatments are not associated with better survival than current strategies, they would be beneficial if their costs were not higher than half of this over-cost. If such CMV prophylaxis improves overall survival, it should be cost-effective.

Our study has limitations: (1) it is monocentric. However, our center is representative of French practice and all our patients were managed according to common procedures, closely followed in one center, such that we did not miss any significant complication. This design with a systematic check of CMV episodes in the original medical charts is more precise than large studies based on CMV coding in healthcare databases which may have underestimated the CMV episodes when not coded [32] (2) We did not take into consideration non-viral infections that are often associated with GVHD and CMV episodes. However, most fungal or bacterial infections in such patients require hospitalization and are, thus, indirectly captured in the length of stay. (3) During the study period and until now, we did not use valganciclovir, the oral pro-drug of ganciclovir, for first line pre-emptive therapy of CMV infection. Although associated with hematological toxicity and especially neutropenia which is a limiting factor to its prolonged administration [33], the use of valganciclovir may facilitate outpatient management and should reduce costs, but is not recommended as an “AI” first line treatment of CMV infection after stem cell transplant, especially in high risk patients [20]. Despite these limitations, our study is the largest cost study so far run in Europe, and while most cost analyses are commissioned by drug companies [34], our study is academic.

## Conclusion

In summary, the costs of allogeneic HSCT increase by 25–30% when there are CMV episodes. The cost difference between patients who develop CMV episodes and those who do not could be reduced by appropriate use of prophylactic strategies.

## Abbreviations

CMV: Cytomegalovirus
D: Donor
DRG: Diagnostic related groups
EBMT: European Group for Blood and Marrow Transplantation
GVHD: Graft-versus-host disease
HSCT: Hematopoietic Stem Cell Transplantation
ICU: Intensive Care Unit
PCR: Polymerase chain reaction
qPCR: Quantitative PCR
R: Recipient
SD: Standard deviation
